# Small RNAs: Efficient and miraculous effectors that play key roles in plant–microbe interactions

**DOI:** 10.1111/mpp.13329

**Published:** 2023-04-07

**Authors:** Chun‐Hao Jiang, Zi‐Jie Li, Li‐Yu Zheng, Yi‐Yang Yu, Dong‐Dong Niu

**Affiliations:** ^1^ Department of Plant Pathology, College of Plant Protection Nanjing Agricultural University Nanjing China; ^2^ Key Laboratory of Integrated Management of Crop Disease and Pests, Ministry of Education/Key Laboratory of Integrated Pest Management on Crops in East China, Ministry of Agriculture/Key Laboratory of Plant Immunity Nanjing Agricultural University Nanjing China; ^3^ Engineering Center of Bioresource Pesticide in Jiangsu Province Nanjing China

**Keywords:** cross‐kingdom trafficking, plant immunity, RNA interference (RNAi), plant–pathogen interaction, small RNA

## Abstract

Plants' response to pathogens is highly complex and involves changes at different levels, such as activation or repression of a vast array of genes. Recently, many studies have demonstrated that many RNAs, especially small RNAs (sRNAs), are involved in genetic expression and reprogramming affecting plant–pathogen interactions. The sRNAs, including short interfering RNAs and microRNAs, are noncoding RNA with 18–30 nucleotides, and are recognized as key genetic and epigenetic regulators. In this review, we summarize the new findings about defence‐related sRNAs in the response to pathogens and our current understanding of their effects on plant–pathogen interactions. The main content of this review article includes the roles of sRNAs in plant–pathogen interactions, cross‐kingdom sRNA trafficking between host and pathogen, and the application of RNA‐based fungicides for plant disease control.

## Graphical abstract

This review summarizes the effects of small RNAs (sRNAs) on plant–pathogen interactions and the recent discoveries of cross‐kingdom sRNA trafficking, and discusses the possibility of using RNA‐based fungicides for plant protection.

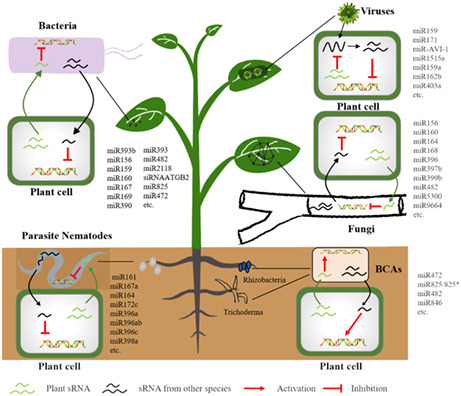

## 1 INTRODUCTION

Plants are constantly under attack by many pathogens and pests, causing devastating food and economic losses worldwide (Schaal, 2019; Wang, Thomas, et al., 2017). To survive in a complex and hostile soil environment, plants have evolved multiple types of inducible immune responses to attacks by pathogens (Jiang, Fan, et al., 2020; Niu et al., 2016). In recent decades, it has been demonstrated that plants have evolved a complex immune system, which consists of two major branches. One is called pathogen‐associated molecular pattern (PAMP)‐triggered immunity (PTI), which recognizes conserved microbial PAMPs, such as flagellin, chitin, and glycoprotein, using membrane‐bound receptors (pattern recognition receptors, PRRs) or surface receptors (transmembrane receptor‐like kinases) (Jiang, Fan, et al., 2020; Hua et al., 2018; Schwessinger & Zipfel, 2008). PTI is usually accompanied by induction of pathogenesis‐related (PR) gene expression, production of reactive oxygen species (ROS), callose deposition, and salicylic acid (SA) accumulation (Jwa & Hwang, 2017; Withers & Dong, 2017). Pathogens, in turn, secrete effector proteins into host plants to suppress PTI, promoting successful infection and causing disease (Hua et al., 2018; Thomma et al., 2011). Some plants have evolved a second type of immune response to inhibit pathogen invasion, called effector‐triggered immunity (ETI). ETI acts largely inside the plant cell via polymorphic proteins containing a nucleotide‐binding (NB) domain and a leucine‐rich repeat (LRR) structure, which are encoded by plant disease resistance (R) genes (Kong et al., 2017).

For the plant, the successful initiation of the innate immune response on pathogen infection requires comprehensive and accurate gene expression reprogramming and communication between the host and microorganisms. Recently, several investigations have shown that many small RNAs (sRNAs) are involved in genetic expression and reprogramming affecting plant–pathogen interactions (Huang et al., 2019). Plant sRNAs (18–30 nucleotides [nt] in length) can be classified into two major categories, termed microRNAs (miRNA) and small interfering RNAs (siRNA), according to their biogenetic pathways and morphology (Achkar et al., 2016; Cui et al., 2017; D'Ario et al., 2017). There are also further special classes, such as *trans*‐acting small interfering RNAs (ta‐siRNAs), small nuclear RNA (snRNA, also referred to as U‐RNA), natural antisense small interfering RNAs (nat‐siRNAs), long siRNAs (lsiRNAs), and small nucleolar RNA (snoRNA) (Huang et al., 2016; Katiyar‐Agarwal & Jin, 2010; Shahid et al., 2018). The miRNAs are generated from single‐stranded RNAs (ssRNA) with imperfectly base‐paired stem‐loop structures; the siRNAs are generated from long double‐stranded RNAs (dsRNAs) and by RNA‐dependent RNA polymerase (RDR) activity (D'Ario et al., 2017; Devert et al., 2015; Islam et al., 2017; Katiyar‐Agarwal & Jin, 2010). Recently, it was found that the DICER‐LIKE PROTEIN 3 (DCL3) produces 24‐nt siRNAs that determine the specificity of the RNA‐directed DNA methylation pathway. The 24‐nt siRNA length dependence is critical for the separation between the 5′‐phosphorylated end of the guide RNA and dual cleavage sites formed by the paired ribonuclease III domains. The machinery for RNA interference (RNAi) consists of three core components: RDRs for biosynthesis of dsRNA from an ssRNA; DCL, for cleaving ssRNA with imperfectly base‐paired stem‐loop structures or dsRNA into sRNAs; and Argonaute (AGO) proteins, binding sRNAs to form RNA‐induced silencing complexes (RISC) for leading the target mRNA to cleavage or translation suppression (Elbashir et al., 2001; Islam et al., 2018; Zhu et al., 2019). The mechanism of cross‐kingdom RNAi has also been considered and studied in plant–pathogen interactions (Kulshrestha et al., 2020; Weiberg et al., 2013; Zotti et al., 2018). Recently, studies have discovered that sRNAs function as pathogen effectors to regulate host immunity and pathogen infection by silencing target genes in the host (Wang et al., 2016; Weiberg et al., 2014; Weiberg & Jin, 2015). However, a large number of scientific problems behind this mechanism still need to be studied and expounded.

In this review, we summarize the effects of sRNA on plant–pathogen interactions and highlight the recent discoveries of cross‐kingdom sRNA trafficking between host and pathogen. Finally, we also discuss the possibility of using RNA‐based fungicides for plant protection.

## 2 THE ROLE OF sRNAs IN PLANT–PATHOGEN INTERACTIONS

**FIGURE 1:**
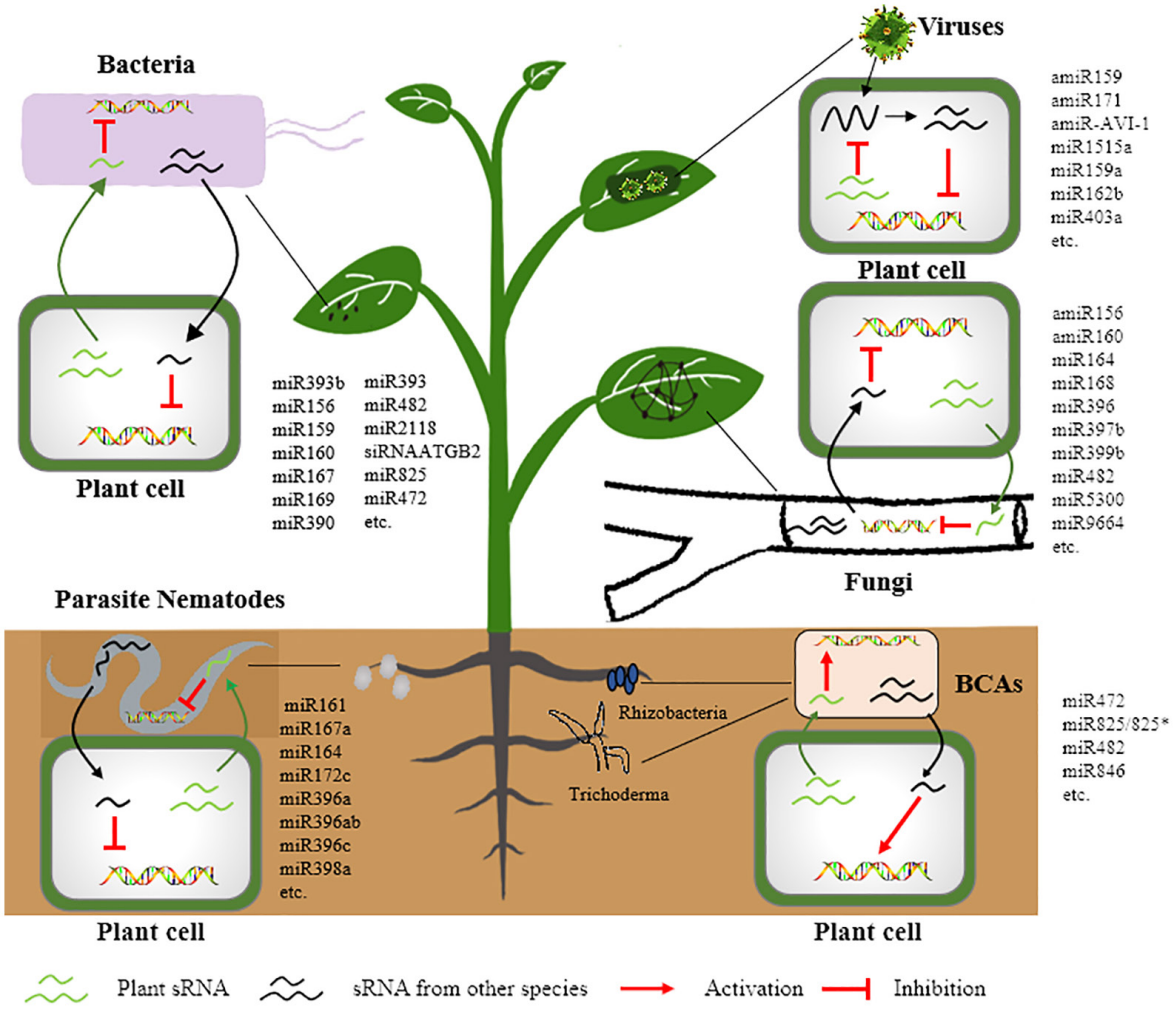
The role of sRNAs against infection by various pathogens.

Various plant diseases caused by pathogens, including oomycetes, fungi, bacteria, viruses, nematodes, mycoplasma, viroids, and other parasites, have caused great damage to crop production and resulted in huge economic losses (Figure 1) (Islam et al., 2018). A number of plant endogenous sRNAs are involved in plant–pathogen interactions and regulation of the immune responses. It has been demonstrated that sRNAs are involved in the plant defence response through different pathways that actively regulate plant immunity to pathogen infection by tackling PAMPs and effectors. The first miRNA found to be involved in plant immunity is the well‐known miR393, which is induced by flg22 (a PAMP); it activates the PTI by silencing the auxin receptors to affect the auxin signalling pathway in *Arabidopsis* (Huang et al., 2019; Navarro et al., 2006). The first sRNA found to be involved in plant immunity was nat‐siRNAATGB2, which is specifically and highly induced by *Pseudomonas syringae* pv. *tomato* (Pst) carrying the effector AvrRpt2; it promotes ETI by silencing a pentatricopeptide repeat‐like protein (a negative regulator of plant defence) (Huang et al., 2019; Katiyar‐Agarwal & Jin, 2010). Table 1 shows the sRNAs involved in plant–pathogen interactions and regulation of immune responses to a variety of pathogens.

**TABLE 1:** List of sRNAs involved in plant–pathogen interactions

miRNAs	Defensive role in plant species	Name of pathogen	Pathogen type	Target	Reference
miR156	*Brassica oleracea*	*Xanthomonas campestris* pv. *campestris*	Bacterium	*ARF*	Santos et al. (2019)
miR159	*Arabidopsis thaliana*	*Pseudomonas syringae*	Bacterium	*MYB33, MYB65, MYC101*	Zhang, Gao, et al. (2011)
miR160	*Arabidopsis thaliana*	*Pseudomonas syringae*	Bacterium	*ARF10, ARF16, ARF17*	Li et al. (2010)
miR167	*Brassica oleracea*	*Xanthomonas campestris* pv. *campestris*	Bacterium	*ARF*	Santos et al. (2019)
miR167	*Arabidopsis thaliana*	*Pseudomonas syringae*	Bacterium	*ARF8, ARF6*	Fahlgren et al. (2007); Zhang, Gao, et al. (2011)
miR169	*Brassica oleracea*	*Xanthomonas campestris* pv. *campestris*	Bacterium	*ARF*	Santos et al. (2019)
miR390	*Brassica oleracea*	*Xanthomonas campestris* pv. *campestris*	Bacterium	*ARF*	Santos et al. (2019)
miR390	*Arabidopsis thaliana*	*Pseudomonas syringae*	Bacterium	*TAS3*	Zhang, Gao, et al. (2011)
miR393	*Arabidopsis thaliana*	*Pseudomonas syringae*	Bacterium	*TIR1, AFB2, AFB3*	Fahlgren et al. (2007); Navarro et al. (2006)
miR393*	*Arabidopsis thaliana*, *Nicotiana benthamiana*	*Pseudomonas syringae*	Bacterium	*MEMB12*	Zhang, Zhao, et al. (2011)
miR398	*Arabidopsis* *thaliana*	*Pseudomonas syringae*	Bacterium	*COX5b.1, CSD1, CSD2*	Jagadeeswaran et al. (2009); Li et al. (2010)
miR408	*Arabidopsis thaliana*	*Pseudomonas syringae*	Bacterium	Copper protein plantacyanin and copper ion‐binding protein genes	Zhang, Gao, et al. (2011)
miR472	*Arabidopsis thaliana*	*Pseudomonas syringae* pv. *tomato*	Bacterium	CC‐NBS‐LRR	Jiang, Fan, et al. (2020)
miR773	*Arabidopsis thaliana*	*Pseudomonas syringae*	Bacterium	*MET2*	Li et al. (2010)
miR825	*Arabidopsis thaliana*	*Pseudomonas syringae*	Bacterium	Remorin, zinc finger homeobox family, frataxin‐related	Fahlgren et al. (2007)
miR1447	*Populus beijingensis*	*Dothiorella gregaria*	Fungus	Disease resistance protein	Chen et al. (2012)
miR1448	*Populus* sp.	*Botryosphaeria dothidea*	Fungus	S‐conjugate, ABC transporter, ATP‐binding cassette transport protein	Lu et al. (2008)
miR1448	*Gossypium hirsutum*	*Verticillium dahliae*	Fungus	Disease resistance protein	Yin et al. (2012)
miR1448	*Populus trichocarpa*	*Botryosphaeria dothidea*	Fungus	Disease resistance protein (NBS‐LRR protein)	Zhao et al. (2012)
miR1450	*Populus trichocarpa*	*Botryosphaeria dothidea*	Fungus	Disease resistance protein (NBS‐LRR protein)	Zhao et al. (2012)
miR156	*Triticum aestivum*	*Erysiphe graminis*	Fungus	*Squamosa‐promoter binding protein‐like*	Nair et al. (2020)
miR160	*Pinus taeda*	*Cronartium quercuum* f. sp. *fusiforme*	Fungus	Auxin response factor, Aux/IAA	Sun (2012)
miR160	*Populus trichocarpa*	*Botryosphaeria dothidea*	Fungus	Auxin response factor, Aux/IAA	Zhao et al. (2012)
miR164	*Gossypium hirsutum*	*Verticillium dahliae*	Fungus	*NAC100*	Hu, Lei, et al. (2020)
miR164a	*Oryza sativa*	*Magnaporthe oryzae*	Fungus	*NAC60*	Wang, Xia, et al. (2018)
miR168	*Brachypodium distachyon*	*Magnaporthe oryzae*	Fungus	*AGO1*	Zanini et al. (2021)
miR396	*Arabidopsis thaliana*	*Plectosphaerella cucumerina*, *Fusarium oxysporum* f. sp. *conglutinans*, *Colletotrichum higginsianum, Botrytis cinerea*	Fungus	*GRF*	Soto‐Suárez et al. (2017)
miR396a‐5p	*Nicotiana tabacum*	*Phytophthora nicotianae*	Oomycete	*GRF*	Chen et al. (2015)
miR397b	*Malus hupehensis*	*Botryosphaeria dothidea*	Fungus	Lignin biosynthesis	Yu et al. (2020)
miR399b	*Triticum aestivum*	*Puccinia striiformis* f. sp. *tritici*	Fungus	*CLP1*	Ramachandran et al. (2020)
miR482	*Gossypium hirsutum*	*Verticillium dahliae*	Fungus	Disease resistance protein	Yin et al. (2012)
miR482a	*Solanum lycospersicum*	*Phytophthora infestans*	Oomycete	lncRNA15492, NBS‐LRR	Jiang, Cui, et al. (2020)
miR482b	*Solanum lycospersicum*	*Phytophthora infestans*	Oomycete	lncRNA23468, NBS‐LRR	Jiang et al. (2018, 2019)
miR5300	*Solanum lycopersicum*	*Fusarium oxysporum*	Fungus	Solyc05g008650, *tm‐2*	Ouyang et al. (2014)
miR9664	*Triticum aestivum*	*Puccinia striiformis* f. sp. *tritici*	Fungus	*CLP1*	Ramachandran et al. (2020)
miRln20	*Malus domestica*	*Glomerella cingulata*	Fungus	*TN1‐GLS*	Zhang, Zhang, et al. (2019)
miRNA‐uniq‐133	*Triticum aestivum*	*Zymoseptoria tritici*	Fungus	*TraesCS6A01G222300.1*	Ma et al. (2020)
miR156	*Solanum lycospersicum*	*Pochonia chlamydosporia*	Mutualistic microbe	*SPL*	Pentimone et al. (2018)
miR167	*Solanum lycospersicum*	*Pochonia chlamydosporia*	Mutualistic microbe	*ARF*	Pentimone et al. (2018)
miR168	*Solanum lycospersicum*	*Pochonia chlamydosporia*	Mutualistic microbe	*AGO1*	Pentimone et al. (2018)
miR10186	*Glycine max*	*Heterodera glycines*	Nematode	*Rhg4*	Lei et al. (2019)
miR10187	*Glycine max*	*Heterodera glycines*	Nematode	*Rhg4*	Lei et al. (2019)
miR10193	*Glycine max*	*Heterodera glycines*	Nematode	*Rhg4*	Lei et al. (2019)
miR393	*Arabidopsis thaliana*	*Phytophthora capsici*	Oomycete	*TIR1*	Hou et al. (2019)
miR2118	*Solanum lycospersicum*	*Phytophthora infestans,* *Pseudomonas syringae* pv. *tomato*	Oomycete, bacterium	*NLR*	Canto‐Pastor et al. (2019)
miR482	*Solanum lycospersicum*	*Phytophthora infestans, Pseudomonas syringae* pv. *tomato*	Oomycete, bacterium	NLR	Canto‐Pastor et al. (2019)
amiR159	*Arabidopsis thaliana*	*Turnip yellow mosaic virus*	Virus	*P69, HC‐Pro*	Niu et al. (2006)
amiR171	*Nicotiana tabacum*	*Caulifower mosaic virus*	Virus	*2b*	Qu et al. (2007)
amiR‐ACI‐1	*Solanum lycospersicum*	*Tomato leaf curl virus*	Virus	*AVI*, *AV2*	Sharma and Prasad (2020)
miR1515a	*Glycine max*	*Soybean mosaic virus*	Virus	*‐*	Bao et al. (2018)
miR159a	*Nicotiana benthamiana*	*Plum pox virus*	Virus	*P1, HC‐Pro*	Simon‐Mateo and Garcia (2006)
miR159a	*Nicotiana tabacum*	*Potato virus Y*	Virus	*HC‐Pro*	Ai et al. (2011)
miR162b	*Glycine max*	*Soybean mosaic virus*	Virus	*‐*	Bao et al. (2018)
miR167b	*Nicotiana benthamiana*	*Plum pox virus*	Virus	*P2, HC‐Pro*	Simon‐Mateo and Garcia (2006)
miR167b	*Nicotiana tabacum*	*Potato virus X*	Virus	*TGBp1, p25*	Ai et al. (2011)
miR168a	*Glycine max*	*Soybean mosaic virus*	Virus	*‐*	Bao et al. (2018)
miR171	*Nicotiana benthamiana*	*Cucumber green mottle virus*	Virus	*‐*	Liang et al. (2019)
miR171a	*Nicotiana benthamiana*	*Plum pox virus*	Virus	*P3, HC‐Pro*	Simon‐Mateo and Garcia (2006)
miR171a	*Nicotiana tabacum*	*Potato virus X* (PVX)	Virus	*TGBp1,p25*	Ai et al. (2011)
miR1885	*Brassica napus*	*Turnip mosaic virus*	Virus	TIR‐NBS‐LRR	Cui et al. (2020)
miR395	*Triticum* *aestivum*	*Wheat streak mosaic virus*	Virus	Conserved region of virus	Fahim et al. (2012)
miR403a	*Glycine max*	*Soybean mosaic virus*	Virus	*‐*	Bao et al. (2018)
miR403a	*Nicotiana benthamiana*	*Tobacco mosaic virus*	Virus	*AGO2*	Diao et al. (2019)
miR482	*Solanum lycopersicum*	*Turnip crinkle virus*, *Cucumber mosaic virus*, *Tobacco rattle virus*	Virus	NBS‐LRR	Shivaprasad et al. (2012)
miR6019	*Solanum lycopersicum*, *Nicotiana tabacum*	*Tobacco mosaic virus*	Virus	*N*	Deng et al. (2018)
miR6020	*Solanum lycopersicum*, *Nicotiana tabacum*	*Tobacco mosaic virus*	Virus	*N*	Deng et al. (2018)

### 2.1 sRNAs in bacteria–plant interactions

Recently, many sRNAs that directly take part in the response to bacterial diseases have been identified (Table 1 and Figure 1). miR393b*/MEMB12 are important effectors or regulators in plant antibacterial immunity (Zhang, Zhao, et al., 2011). In *Arabidopsis*, miR393b* (the complementary strand of miR393) has been identified as an AGO2‐bound sRNA, which could target MEMB12 encoding a SNARE protein localized in Golgi apparatus. Both miR393b* overexpression and *memb12* mutation promoted the secretion of PR1 in *Arabidopsis* in response to Pst DC3000 infection (Zhang, Zhao, et al., 2011). A large number of studies have implicated miR393 as being strongly involved in ETI. It was also found that miR393 was significantly repressed, resulting in the target gene LecRLK (lectin receptor‐like kinase) being up‐regulated to enhance perception ability of bacterial lipopolysaccharide in *Arabidopsis* (Djami‐Tchatchou & Dubery, 2015). The overexpression and repression of miR393, respectively, suppressed and induced the expression of LecRLK in *Arabidopsis* treated with lipopolysaccharide (Djami‐Tchatchou & Dubery, 2019). Repression of auxin signalling constitutes part of a plant's defence response to bacterial infection (Gao & Jin, 2011; Navarro et al., 2006; Pruss et al., 2008). It was observed that miR160 and miR167 were induced in response to Pst DC3000 *hrcC* ^*−*^ and flg22, and that miR160a‐overexpressing plants increased callose deposition after treatment with Pst DC3000 *hrcC* ^*−*^ and flg22 (Yao et al., 2013). Moreover, investigation of the tumours caused by infection of *Agrobacterium tumefaciens* revealed that miR167 and miR393 were significantly down‐regulated and that mutants of these miRNAs showed hypersusceptibility to the bacterium (Li et al., 2010). The miR398 production, targeting *CSD1*, *CSD2* (copper superoxide dismutases) and *COX5b‐1* (a cytochrome coxidase subunit V), was reduced in plants challenged with avirulent strains such as Pst DC3000 *avrRpm1* and Pst DC3000 *avrRpt2* (Jagadeeswaran et al., 2009). It was also found that flg22 suppresses miR398b accumulation. In contrast, the expression of the miR398 target genes *COX5b‐1*, *CSD1*, and *CSD2* is increased (Li et al., 2010). Significantly suppressed miR398 was also observed in citrus plants infected with pathogenic bacteria of the genus “*Candidatus* Liberibacter” (Zhao et al., 2013). Researchers have screened various other miRNA families that are involved in antibacterial defence in plants by deep‐sequencing. For example, Zhang et al. described the expression of 20 diverse miRNA families on the invasion of Pst DC3000 in *Arabidopsis*; most of the target genes were involved in the synthesis and signalling pathways of various hormones such SA, jasmonic acid (JA), and abscisic acid (ABA) (Zhang, Gao, et al., 2011). The involvement of hormone pathways such as SA, JA, and ABA in host defence has been well studied. Thus, these studies show that the miRNAs normally facilitate the fine‐tuning of defence responses rather than targeting some genes involved in the plant immune system directly (Ballaré, 2015; Berens et al., 2017; Ludwig‐Müller, 2015; Qi et al., 2012; Ramegowda & Senthil‐Kumar, 2015; Sanchez‐Vallet et al., 2012; Song et al., 2013; Tamaoki et al., 2013).

Like miRNA, siRNAs have also been reported to be involved in the interaction between plants and bacteria, such as nat‐siRNA, nat‐siRNAATGB2, and some lsiRNAs (Islam et al., 2017; Weiberg & Jin, 2015). Five lsiRNAs were induced in plants against Pst *avrRpt2* infection and an endogenous siRNA siRNAATGB2 was identified that is derived from the natural antisense transcripts pair ATGB2‐PPRL, functioning for plant resistance to Pst *avrRpt2* (Katiyar‐Agarwal et al., 2006, 2007). The activation of secondary siRNA production and amplification of silencing signals is dependent on RDR6. An miR472‐RDR6 silencing pathway has been reported that is required for enhancing plant defence against *P. syringae*. The miR482/2118 family suppresses NB‐LRRs by production of short tandem target mimic RNAs, dependent on RDR6, to enhance plant resistance to *P. syringae* in tomato (Boccara et al., 2014; Canto‐Pastor et al., 2019). Taken together, this suggests that RDR6‐dependent siRNAs are critical regulators of innate immune receptors and modulate plant broad‐spectrum resistance.

Initially, research was focused on endogenous sRNAs in plants that are involved in the host defence response to bacteria. Recent studies have demonstrated that pathogen‐derived sRNAs play an active part in the virulence of pathogens. In bacteria, regulation of efficiency during translation and targeted mRNA stability are modulated by heterogeneous sRNAs. sRNAs involved in regulation of pathogenicity have been identified in *Agrobacterium*, *Pectobacterium*, and *Xanthomonas* (Weiberg et al., 2014). Noncoding sRNAs (sX12 and sX13) were relatively more important for pathogenicity in *Xathomonas campestris* pv. *vesicatoria* invasion: the sRNA sX12 could regulate the expression of HrpX, a type III secretion system (T3SS) regulator, while the sRNA sX13 could modulate the transcription of *HrpF*, *HrcJ*, and *HrcN* genes (Islam et al., 2017; Schmidtke et al., 2012, 2013).

Here, the involvement of sRNAs in bacteria–pathogen interactions is summarized. Although miRNAs or siRNAs are vital components of various defence‐related pathways, the specificity function and contributions on targets of these sRNAs still need to be explored.

### 2.2 sRNAs in fungus/oomycete–plant interactions

Fungi, such as *Botrytis*, *Fusarium*, and *Verticillium,* are the dominant causal agents of plant diseases. To effectively resist fungal infection, plants have evolved immune mechanisms. There is reason to believe that a large number of sRNAs are involved in the process of fungus–pathogen interactions. With high‐throughput sequencing technology and advancements in bioinformatics, the roles of many sRNAs in fungal invasion and development in the host plant have been clarified (Table 1 and Figure 1). Yin et al. conducted comprehensive identification studies of miRNAs and other sRNAs from two cotton cultivars, Hai‐7124 and Yi‐11, that showed resistance and susceptibility to *Verticillium*, respectively (Yin et al., 2012). Among all the documented miRNA families, over 65 miRNAs that presented modified expression in response to *Verticillium* infection in the cotton cultivars were identified. Three specific miRNAs, Ptc‐miR482, Ptc‐miR1444, and Ptc‐miR1448, were found to regulate plant biotic and abiotic stress tolerance by targeting polyphenol oxidase genes and disease resistance‐related genes (*R* genes) (Lu et al., 2008). A recent study has shown that specific miRNAs and target gene cross‐talk are involved in cotton resistance to Verticillium wilt. To understand this, sRNA libraries were constructed from mock‐infected and *Verticillium*‐infected roots of two cotton cultivars. Deep sequencing identified a total of 383 miRNAs and determined that two miRNAs, GhmiR165 and GhmiR395, were possibly involved in the response to *Verticillium dahliae* by regulating the establishment of the vascular pattern and secondary cell wall formation through the GhmiR165‐REV module and by affecting sulphur assimilation through the GhmiR395‐APS1/3 module (Mei et al., 2022). The GhmiR477‐CBP60A was also characterized as involved in the late response of cotton to *V. dahliae* infection. GhmiR477 directly cleaved the mRNA of the *GhCBP60A* gene in the posttranscriptional processing. GhmiR477‐silencing decreased plant resistance to *V. dahliae* infection while the knockdown of *GhCBP60A* increased plant resistance to the pathogen (Hu, Hao, et al., 2020). sRNA also plays an important role in the interaction between oomycetes and host plants. The oomycete *Phytophthora* can reprogramme host pre‐mRNA splicing to subvert immunity; the *Phytophthora* effector PSR1 targets a novel component of the sRNA pathway in plants to promote infection (Gui et al., 2022; Huang et al., 2017; Qiao et al., 2015). The WY domain in PSR1 is required for infection and RNA silencing suppression activity (Zhang, Jia, et al., 2019).

Deep sequencing of sRNA libraries from susceptible and resistant rice lines uncovered the important role that sRNAs play in enhancing immunity against rice blast disease caused by *Magnaporthe oryzae* (Li et al., 2014). miR160a and miR398b overexpression lines showed resistance to *M. oryzae* by increasing hydrogen peroxide accumulation and raising the expression of pathogenicity‐related genes (*PR* genes) to decrease fungal growth in the rice plants (Li et al., 2014). It was also found that Osa‐miR7695 overexpression resulted in plant resistance to *M. oryzae* and thus that Os‐miR7695 modulated plant immunity, illustrating a novel regulatory network targeting natural resistance‐associated macrophage protein 6 (OsNramp6) (Campo et al., 2013). RNAi is conserved in eukaryotic organisms, and the sRNAs and their biogenesis in the context of growth and pathogenicity of *M. oryzae* have also been studied recently. The loss of a single gene encoding Dicer, RNA‐dependent RNA polymerase, or Argonaute, which are each required for the biogenesis of sRNA‐matching genome‐wide regions, reduces the sRNA level in *M. oryzae*. Moreover, the loss of one Argonaute gene reduced both sRNA and the virulence of *M. oryzae* on barley leaves (Raman et al., 2017). The sRNA236 was identified as a microRNA‐like milR236 that targets a histone acetyltransferase type B catalytic subunit (MoHat1), which is involved in appressorium function and virulence. Overexpression of milR236 results in delayed appressorium formation and virulence attenuation, phenotypes that are similar to Δ*Mohat1* mutant strain (Li et al., 2020). Rice sheath blight, caused by the necrotrophic pathogen *Rhizoctonia solani,* is considered to be one of the most devastating rice diseases worldwide; sRNAs also play an important role in the interaction between rice and *R. solani*. By using deep sequencing, rice lsiRNAs were found to be a unique class of endogenous sRNAs produced in rice, and may participate in the response against *R. solani*. A group of rice lsiRNAs, in the range of 25–40 nt in length, have been identified and some of these rice lsiRNAs are differentially expressed on infection of *R. solani*. Rice lsiRNAs require OsDCL4 for biogenesis and OsAGO18 for function (Niu et al., 2017). Other studies have found that 468 known mature miRNAs and 747 putative novel miRNAs may be involved in rice–*R. solani* interactions (Srikakulam et al., 2022). The rice sRNA expression patterns against *R. solani* were screened; MITE‐derived siRNA siR109944 expression was significantly suppressed on *R. solani* infection. siR109944 has a conserved function in interfering with plant growth, development, and immunity to *R. solani* by affecting auxin homeostasis (Qiao et al., 2020). We know that the expression of miRNAs also differs in the different growth stages of fungi; for example, miRNAs identified from the hyphae and microconidia of *Fusarium oxysporum* show differential expression levels. Fon‐miR7696a‐3p and Fon‐miR6108a were identified to modulate the enhancement of the biosynthesis of the toxin‐related gene in *F. oxysporum* (Jiang et al., 2017; Kulshrestha et al., 2020).

Phytohormones play a major role in plant defence against pathogen attack, including fungal pathogens. Some fungal pathogens mimic the function of phytohormones, which enables them to manipulate the regulation of signalling in plant defence, resulting in hormonal imbalance and impacting the defence response (Fonseca et al., 2018; Kulshrestha et al., 2020). However, plants have evolved a series of measures to down‐regulate some hormones when attacked, to prevent pathogens from using them as virulence factors. For example, some miRNAs can act as regulators to modulate the expression of functional genes in signalling pathways. It has been demonstrated that miR393 is essential for the auxin‐mediated response by down‐regulating the transport inhibitor response 1 (TIR1) during powdery mildew infection in wheat (Nowara et al., 2010).

### 2.3 sRNAs in virus– or viroid–plant interactions

Plants may suffer from viruses and viroids, resulting in reduced yield and economic losses. Some of the best‐studied viruses in crop plants are tobacco mosaic virus (TMV), tomato spotted wilt virus (TSWV), and tomato yellow leaf curl virus (TYLCV). Plants' immunity against viruses is mainly provided by posttranscriptional gene silencing (PTGS), triggered in response to viral/viroid infection to suppress viral replication and spread. Recent studies have shown that sRNAs play essential roles in the interactions between plants and viruses/viroids (Figure 1). sRNAs are involved in PTGS transgene silencing in plants; the sRNAs are complementary to the sense transcript of the transgene (Hamilton & Baulcombe, 1999; Zhao et al., 2016). Recently, a diverse array of virus‐responsive sRNAs has been identified during plant–virus interactions. It was demonstrated that bra‐miR158 and bra‐miR1885, which are specific to *Brassica rapa*, were greatly up‐regulated in response to turnip mosaic virus infection, and the mechanism of bra‐miR158 and bra‐miR1885 regulating plant immunity by targeting TIR‐NBS‐LRR was clarified (Hewezi et al., 2008). In addition, the expression level of miR482 decreased, which in turn relieved some NBS‐LRR proteins to activate ETI in tomato challenged with turnip crinkle virus, tobacco rattle virus, or cucumber mosaic virus (Prasad et al., 2019; Shivaprasad et al., 2012). miR6019/6020 is another class of miRNAs that can target R genes, specifically the tobacco *N* gene that confers resistance to TMV, and produces phased siRNAs in the normal state, which represses the R gene by the cleavage of transcripts in *Nicotiana benthamiana* (Li et al., 2012). miR159, miR172, and miR319 play an important role in plant immunity against virus infection, targetting Myb, AP2, and TCP transcription factors, respectively, to respond to tomato leaf curl New Delhi virus infection (Naqvi et al., 2010). Recently, a study showed that miR171b is involved in the rice–rice stripe virus interaction, as miR171b was down‐regulated in rice when infected by this virus. Moreover, transgenic plants overexpressing miR171b showed more susceptibility to the virus, whereas the opposite response was observed in the miR171b target mimic lines (Tong et al., 2017). Although many sRNAs have been reported to be involved in plant–virus interactions, the molecular mechanism of sRNAs response to viral infections is not yet known. It will therefore be interesting to investigate the role of RNA in viral infections and the pathways associated with the observed response in the future.

Viroids are small (250–400 nt) single‐stranded, circular RNA, pathogens, and infect several crop plants causing diseases of economic importance (Navarro et al., 2021). Viroids are known to initiate a range of sRNAs in plants. For example, RNA silencing is targeted and activated by potato spindle tuber viroid (PSTVd) in infected potato plants (Dalakouras et al., 2015). Some viroids, such as avocado sun blotch viroid and chrysanthemum chlorotic mottle viroid, replicate in the chloroplast. cPeach latent mosaic viroid can trigger the production of vdsiRNAs (St‐Pierre et al., 2009). The generation of vdsiRNAs from both the positive and the negative strands of the viroid genome highlights the cardinal processing of vdsiRNAs from the viroid genomic RNA secondary structure. Additional evidence showed that the viroids can undergo sRNA‐mediated degradation (Schwind et al., 2009). Transgenic tomato plants that express inverted repeats of the PSTVd sequence accumulated high levels of hairpin‐derived sRNAs, and these plants were resistant to PSTVd infection. More interestingly, the degree of resistance to PSTVd was directly correlated with accumulation levels of sRNAs. Recently, RNAi‐based strategies used for controlling viroid infections have been demonstrated, including the use of synthetic *trans*‐acting siRNAs and artificial microRNAs (Carbonell & Daròs, 2017; Di Serio et al., 2022; Flores et al., 2017). Thus, this finding provides evidence that engineering viroid resistance for disease control is feasible.

Long noncoding RNAs (lncRNAs) are transcripts of over 200 nt that have no coding potential but act as regulators via a variety of molecular mechanisms. Recently, a study unveiled lncRNAs as new molecular elements in the plant defence response to virus infection (Wang et al., 2015). For example, several lncRNAs were differentially expressed during TYLCV infection; silencing of two of these, lncRNA‐0761 and lncRNA‐0049, resulted in an increase in disease severity (Wang et al., 2015). In another work on the identification of lncRNAs as key regulators of gene expression in the tomato–TYLCV system, RNA‐sequencing revealed different patterns of lncRNAs and circular RNAs (circRNAs) from plants infected with TYLCV compared to healthy plants. Silencing of sly‐lnc0957 resulted in enhanced resistance to TYLCV in susceptible tomato cultivars. In this case, the lncRNA was demonstrated to be a negative regulator of TYLCV infection (Wang, Yang, et al., 2018). Similarly, in response to maize Iranian mosaic virus infection, the maize plants showed different expressions of circRNAs; deep sequencing identified 155 circRNAs were up‐regulated whereas five were down‐regulated. Among these were 23 maize miRNAs that were responsible for regulating plant development and metabolism (Ghorbani et al., 2018). Moreover, cucumber green mottle mosaic virus infection of watermelon results in differential expression of 548 and 67 lncRNAs, which are responsible for phenylalanine metabolism, citrate cycle, and endocytosis (Shrestha & Józef, 2020; Sun et al., 2020). Taken together, all the above results demonstrate the complex nature of lncRNAs and circRNAs in defence signalling pathways and indicate their function in the regulation of defence response genes. Therefore, studying the function of lncRNAs and circRNAs in antiviral immunity will change our understanding of RNA regulation and help to design new antiviral strategies.

### 2.4 sRNAs in nematode–pathogen interactions

Plant‐parasitic nematodes (PPNs) seriously threaten the safety of crop and agriculture production. PPNs can infect a variety of economically important crops like rice, wheat, maize, soybean, potato, tomato, and sugar beet. Over 4300 plant species from 197 genera have so far been reported as hosts of PPNs, and PPNs lead to over $150 billion losses in annual crops globally (Ali et al., 2017, 2019). Recently, RNAi has been demonstrated in PPNs and shown to be influenced by sRNAs. It is known that miRNAs take part in plant–PPN interactions, for example different miRNAs are down‐regulated to resist *Heterodera schachtii*, such as miR161, miR167a, miR164, miR172c, miR396a, miR396ab, miR396c, and miR398a (Hewezi et al., 2017; Khraiwesh et al., 2012). Furthermore, five root‐knot nematode (RKN)‐responsive miRNAs in the JA‐deficient *spr2* tomato mutant line were identified by comparing susceptible and resistant cultivars (Zhao et al., 2015). Some conserved miRNA families have been identified as present with similar expression profiles in galls from different plant species. For instance, the conserved miR159 and miR172 are up‐regulated in *Arabidopsis*, cotton, and tomato galls (Lei et al., 2019). Kaur and associates reported genome‐wide identification and characterization of both tomato and RKN miRNAs simultaneously from RKN‐infected susceptible tomato roots using a high‐throughput sequencing technique (Kaur et al., 2017). In their study, 281 novel miRNAs of tomato, in addition to 52 conserved and four variants of conserved miRNAs, were identified. In addition, a few conserved miRNAs, such as miR156, miR164, miR159, and miR396 and their targets (SBP, NAC, GAMYB‐like, and GRF1 transcription factor) were confirmed by a negative correlation between expression profiles. Furthermore, a novel Sly_miRNA996 showed a negative correlation with its target MYB‐like transcription factor (Kaur et al., 2017). In fact, recent studies have shown that a large number of miRNAs contribute to the acquired immunity against PPN attack through modulating the expression of plant miRNAs. A few studies have revealed that siRNAs are involved in plant–nematode interactions. It was found that the roots of *Arabidopsis* infected with PPNs induced an overexpression of 24 nt siRNA associated with RNA‐directed DNA methylation in galls and that gall‐specific rasiRNAs could target retrotransposons, primarily GYPSY and COPIA (Ruiz‐Ferrer et al., 2018).

### 2.5 sRNAs function in biological control agent‐induced systemic resistance

Plants have a complex network of interactions with many microorganisms. In addition to pathogens, there is also a class of beneficial microorganisms that can interact with plants. Beneficial microorganisms may stimulate plant growth and enhance resistance to disease and abiotic stresses, and such beneficial microorganisms are termed biological control agents (BCAs). Various BCAs have shown potential to induce systemic resistance, such as *Bacillus* spp., *Pseudomonas* spp., *Trichoderma* spp., and arbuscular mycorrhizal fungi, which can stimulate defence responses and help plants to obtain broad‐spectrum disease resistance through modulating the accumulation of phytohormones and the expression of defence regulatory proteins (Yu et al., 2022). Recently, some studies have shown that sRNAs are also involved in the induced systemic resistance triggered by BCAs. For example, *Bacillus cereus* AR156 triggers induced systemic resistance against Pst DC3000 by suppressing miRNA accumulation in *Arabidopsis*. *B. cereus* AR156 suppresses the miR472 and miR825/825*, and activates R gene‐mediated basal immunity (Jiang, Fan, et al., 2020; Niu et al., 2016). Other studies found that *Bacillus amyloliquefaciens* FZB42 represses plant miR846 to induce systemic resistance via a JA‐dependent signalling pathway (Xie et al., 2018).

## 3 CROSS‐KINGDOM RNAi IN PLANT–MICROBE INTERACTIONS

Previous studies showed that most sRNAs function endogenously during the interaction between plants and microorganisms. Recent evidence has shown that some sRNAs can move between the host cell and interacting organisms, and induce gene silencing via a mechanism called “cross‐kingdom RNAi” (Huang et al., 2019). Cross‐kingdom RNAi was first demonstrated in plant–fungus interactions (Weiberg et al., 2013). It found that *Botrytis cinerea* sRNAs (Bc‐sRNAs) could hijack the host RNAi mechanism in *Arabidopsis* and tomato by binding AGO1 and silencing genes involved in immunity. These fungal sRNAs represent a novel class of effectors that can inhibit host immunity with both DCL1 and DCL2 of *B. cinerea*. The *dcl1/dcl2* double mutant lost the ability to produce sRNA effectors and showed a significant reduction in pathogenicity (Zotti et al., 2018). Since then, similar results have been reported and more sRNA effectors have been identified from other pathogens (Wang et al., 2016; Wang, Weiberg, et al., 2017; Wang, Sun, et al., 2017). For example, *B. cinerea* delivers Bc‐siR37 into the host cell to suppress immunity by targeting more than 15 genes, including receptor‐like kinases, WRKY transcription factors, and cell wall‐modifying enzymes. As a result, *At‐WRKY7*, *At‐PMR6*, and *At‐FEI2* exhibited enhanced disease susceptibility to *B. cinerea* (Wang, Weiberg, et al., 2017). Moreover, it was found that the *Arabidopsis ago1‐27* mutant was more resistant to *Verticillium dahliae*, which causes Verticillium wilt disease on many crops. Similar results have also been reported for *B. cinerea*. These results indicate that *V. dahliae* also uses sRNAs to silence host target genes and which are associated with *Arabidopsis* AGO1 during infection (Wang et al., 2016). *Puccinia striiformis* f. sp. *tritici* also delivers a novel microRNA‐like RNA1 (milR1) into wheat host cells and suppresses wheat pathogenesis‐related 2 (*PR2*) gene in the defence pathway. Silencing of the milR1 precursor led to enhanced wheat resistance to *P. striiformis* f. sp. *tritici* (Wang, Sun, et al., 2017). In addition, cross‐kingdom sRNA transport from microbes to hosts is not restricted to eukaryotic pathogens that encode RNAi machinery. For instance, the protozoan parasite *Trypanosoma cruzi* produces tRNA‐derived sRNAs that contribute to the ability to infect mammalian cells, although *T. cruzi* lacks canonical sRNA pathways (Garcia‐Silva et al., 2014). Another study showed that the parasitic plant *Cuscuta campestris* can send miRNAs into host plants to silence host genes involved in the defence responses against *C. campestris* (Shahid et al., 2018). Additionally, the symbiotic bacterium *Rhizobium* delivers tRNA‐derived sRNA fragments into soybean cells in an AGO1‐dependent manner, thus inducing plant nodulation‐related gene silencing as in *B. cinerea* and *V. dahliae* (Ren et al., 2019).

Trans‐kingdom RNA plays a key role in host–parasite interactions. It was recently discovered that animals and plants can deliver host sRNAs into interacting microbes to suppress their virulence by targeting pathogen virulence genes and inhibit their invasion. For example, host sRNAs were identified by Cai et al. (2018) including miRNAs and siRNAs, in purified fungal protoplasts obtained from infected plant tissue. Among these, many of the transported host sRNAs can potentially silence *B. cinerea* genes that are involved in pathogen virulence. These gene mutant strains were found to be much less virulent on host plants (Cai et al., 2018). Moreover, as for *B. cinerea*, the cotton plants can also deliver host sRNAs to *V. dahliae* during infection. Moreover, 28 miRNAs from *V. dahliae* recovered from infected cotton plants were identified (Zhang et al., 2022). It was found that two miRNAs, miR159 and miR166, target the fungal gene isotrichodermin C‐15 hydroxylase (*VdHiC‐15*) and Ca^2+^‐dependent cysteine protease calpain (*VdClp‐1*), respectively (Zhang et al., 2016). It was also shown that *Arabidopsis* plants can deliver siRNAs, secondary phasiRNAs, into *Phytophthora capsici*, an oomycete pathogen, to induce the silencing of genes involved in pathogenicity (Hou et al., 2019).

## 4 APPLICATION OF CROSS‐KINGDOM RNAi IN CROP PROTECTION

**FIGURE 2:**
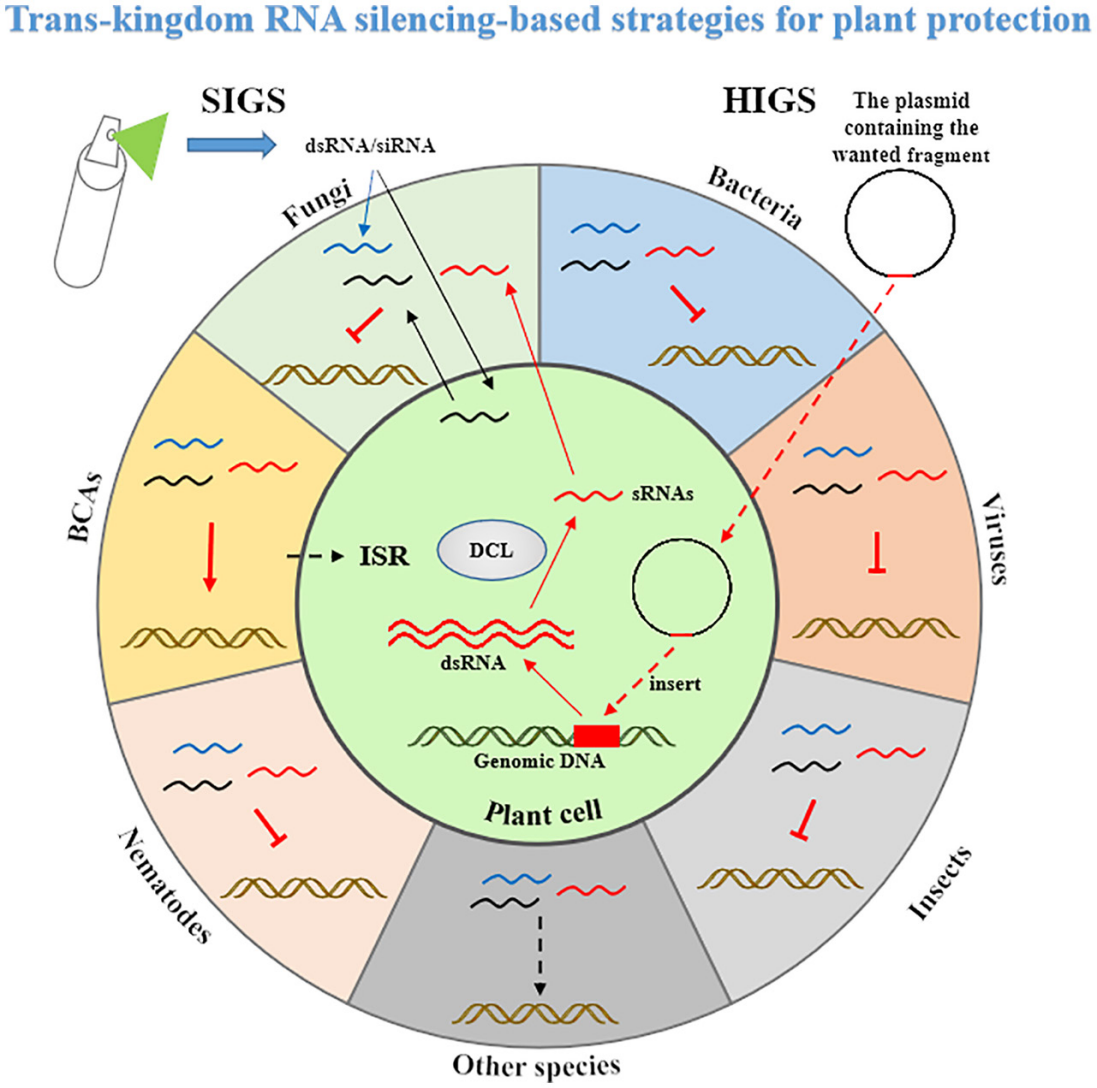
The application of trans‐kingdom RNA silencing to plant disease resistance to pests and pathogens.

The ultimate goal of agricultural basic research is to transform new discoveries and advanced technologies into practical applications. Host‐induced gene silencing (HIGS) technology is an innovative concept of cross‐kingdom RNAi technology that has emerged as a powerful alternative to chemical treatments for crop protection (Figure 2). Numerous studies have demonstrated successful applications of HIGS technology in plants against a wide variety of plant diseases caused by pathogens such as viruses, viroids, bacteria, oomycetes, fungi, nematodes, and pests such as herbivorous insects, which cause significant economic loss (Coleman et al., 2015; Eschen‐Lippold et al., 2012; Escobar et al., 2001; Fairbairn et al., 2007; Ghag, 2017; Govindarajulu et al., 2015; Huang et al., 2006, 2019; Koch et al., 2013; Mao et al., 2007; Niu et al., 2021; Nowara et al., 2010; Panwar et al., 2018; Pooggin et al., 2003; Schwind et al., 2009; Seemanpillai et al., 2003; Shivakumara et al., 2017; Waterhouse et al., 1998; Zhang et al., 2015). The HIGS strategy against plant viruses is an established technology. Virus resistance and gene silencing in plants can be induced by simultaneous expression of sense and antisense RNA (Waterhouse et al., 1998). HIGS has also been demonstrated for DNA viruses, such as tomato leaf curl virus and Vigna mungo yellow mosaic virus, by methylation of the viral promoter sequences (Pooggin et al., 2003; Seemanpillai et al., 2003). Moreover, transgenic tomato plants expressing a hairpin RNA (hpRNA) construct derived from PSTVd sequences exhibit resistance to PSTVd infection and these results provide the possibility for the application of HIGS technology in the prevention and control of viroid diseases (Schwind et al., 2009). In perennial crops, crown gall disease, which is caused by the soil bacterium *Agrobacterium tumefaciens*, results in significant economic losses worldwide. Transgenic *Arabidopsis thaliana* and tomato plants that express two self‐complementary RNA constructs designed to initiate RNAi of *ipt* and *iaaM* were highly resistant to crown gall disease (Escobar et al., 2001). For fungal diseases, HIGS was first reported in the biotrophic powdery mildew fungus, *Blumeria graminis*. It was shown that transgenic barley and wheat that express target‐specific double‐stranded or antisense RNA could inhibit the development of *B. graminis* (Nowara et al., 2010). Recent research has shown that HIGS is also effective in controlling necrotic fungal pathogens such as *V. dahliae*, *B. cinerea*, *Puccinia triticina*, and *Fusarium* species (Koch et al., 2013; Panwar et al., 2018; Wang et al., 2016). Down‐regulation of syntaxin gene expression in potato by HIGS significantly suppressed *Phytophthora infestans* (Panwar et al., 2018). HIGS can also provide effective control of another oomycete disease, downy mildew disease, caused by *Bremia lactucae,* in lettuce. Transgenic lettuce lines expressing inverted repeats of fragments of either the *HAM34* or *CES1* genes of *B. lactucae* resulted in greatly reduced growth and inhibition of sporulation of the pathogen due to the specific suppression of these genes (Govindarajulu et al., 2015). In nematode disease control, HIGS technologies have also been reported recently (Fairbairn et al., 2007; Huang et al., 2006; Shivakumara et al., 2017). Silencing of two pharyngeal gland genes, *msp18* and *msp20*, conferred transcriptional alteration of cell wall‐modifying enzymes in *Meloidogyne incognita* and reduced nematode infectivity in eggplant (Shivakumara et al., 2017). In addition, HIGS of insect growth and development is a promising strategy for pest control in practice (Baum et al., 2007; Coleman et al., 2015; Mao et al., 2007; Zhang et al., 2015). Plant‐mediated RNAi of *MpPIntO2*, *MpC002*, and *Rack1* genes significantly decreased aphid population growth and reduced aphid reproduction by 40%–60% (Coleman et al., 2015). Taken together, all these examples illustrate that HIGS is a promising strategy to limit chemical‐based pesticide applications.

Although HIGS is a promising technology, it relies on the generation of transgenic plants. Transgenic technology has not been successful in some crops, which limits the application of HIGS. Due to the lengthy and costly process of generating transgenic crops, an ecofriendly, non‐genetically modified, RNAi‐based crop protection strategy, spray‐induced gene silencing (SIGS), has been developed. SIGS is a potential, nontransformative, and environment‐friendly pest and pathogen management strategy in which naked or nanomaterial‐bound dsRNA is sprayed onto leaves to cause selective knockdown of pathogenicity genes. It was found that spraying of dsRNA targeting fungal *MoDES1* induced silencing of *MoDES1* in *M. oryzae* and conferred efficient resistance against blast disease (Sarkar & Roy‐Barman, 2021). In addition, SIGS approaches using the application of exogenous dsRNA can also suppress infection of *Brassica napus* by the pathogens *B. cinerea* and *Sclerotinia sclerotiorum* (McLoughlin et al., 2018). Similarly, RNAi‐based control of *Fusarium graminearum* infections through spraying of long dsRNAs has been reported (Koch et al., 2016). Recently, it was reported that the efficacy of SIGS approaches is dependent on the RNA uptake efficiency of the pathogen (Qiao et al., 2021). To improve both RNA uptake efficiency and stability, current research efforts are focused on nanoparticle technology to improve the application system and the limited durability of the RNAi effect (Qiao et al., 2021). To facilitate the effective application of HIGS and SIGS, further studies will be needed to address the underlying mechanisms for cross‐kingdom RNAi between plants and microbes.

## 5 CONCLUSIONS AND FUTURE PERSPECTIVES

Collectively, many studies have highlighted the involvement of sRNAs in plants and pathogens. However, most studies are confined to the computational prediction of sRNA targets, and many still need experimental validation. Moreover, deeper insights into the physiological and molecular roles of sRNAs remain elusive. How are sRNAs transferred in plants and pathogens? How are external RNAs taken up by plants and pathogens? For SIGS application, more mechanisms need to be revealed, for example the stability and absorption efficiency of sRNAs needs to be improved. The new generation of RNAi‐based fungicides should enable an effective strategy for disease and pest control in the future.

## CONFLICT OF INTEREST STATEMENT

The authors declare they have no conflict of interest.

## Data Availability

Data sharing is not applicable to this article as no new data were created.
